# PER2 Regulates Reactive Oxygen Species Production in the Circadian Susceptibility to Ischemia/Reperfusion Injury in the Heart

**DOI:** 10.1155/2021/6256399

**Published:** 2021-10-08

**Authors:** Yaqian Weng, Hui Li, Lin Gao, Wenjing Guo, Shiyuan Xu, Le Li

**Affiliations:** Department of Anesthesiology, Zhujiang Hospital, Southern Medical University, Guangzhou, Guangdong 510282, China

## Abstract

The main objective of this study was to investigate the diurnal differences in Period 2 (PER2) expression in myocardial ischemia-reperfusion (I/R) injury. We investigated diurnal variations in oxidative stress and energy metabolism after myocardial I/R in vitro and in vivo. In addition, we also analyzed the effects of H_2_O_2_ treatment and serum shock in H9c2 cells transfected with silencing RNA against Per2 (siRNA-Per2) in vitro. We used C57BL/6 male mice to construct a model of I/R injury at zeitgeber time (ZT) 2 and ZT14 by synchronizing the circadian rhythms. Our in vivo analysis demonstrated that there were diurnal differences in the severity of injury caused by myocardial infarctions, with more injury occurring in the daytime. PER2 was significantly reduced in heart tissue in the daytime and was higher at night. Our results also showed that more severe injury of mitochondrial function in daytime produced more reactive oxygen species (ROS) and less ATP, which increased myocardial injury. In vitro, our findings presented a similar trend showing that apoptosis of H9c2 cells was increased when PER2 expression was lower. Meanwhile, downregulation of PER2 disrupted the oxidative balance by increasing ROS and mitochondrial injury. The result was a reduction in ATP and failure to provide sufficient energy protection for cardiomyocytes.

## 1. Introduction

Acute myocardial infarction (AMI) is a major cause of morbidity and mortality worldwide. Emergency treatment for AMI has been revolutionized by timely reperfusion therapy [1]. Reperfusion is “a double-edged sword.” It not only provides oxygen and nutrients for ATP production and washes out toxic metabolite accumulation but also induces a burst of reactive oxygen species (ROS) leading to a second injury or dominant injury [2, 3]. However, there is no therapy directly targeting the injury caused by reperfusion.

The frequency of the onset of AMI is not random and has been reported to have a circadian variation, with the peak in the early morning [4]. Also, circadian rhythmicity of the heart has been reported in multiple animal studies [5, 6]. In particular, recent studies using mutant mouse models with cardiomyocyte-specific defects in the core clock machinery suggested that susceptibility of the myocardium itself varies in a time-of-day-specific fashion [7]. However, the molecular mechanism remains poorly understood.

The circadian clock within the heart modulates myocardial metabolism, which in turn facilitates anticipation of diurnal variations in workload, stimuli, and/or the energy supply-to-demand ratio [8]. ROS, as an intrinsic by-product of oxidative phosphorylation, also undergoes circadian rhythms [9]. It may be beneficial for the organism to coordinate catabolism/energy utilization and ROS clearance in a circadian fashion. Nonetheless, a master transcriptional regulator of both processes is not known.

Period 2 (PER2), a molecular component of the mammalian circadian clock, plays a key role in controlling the circadian rhythms in physiology and behavior [10]. Recently, increasing evidence has suggested that PER2 mediates the cardiac protection effect [11, 12]. *Per2*^−/−^ mice have larger infarct sizes with deficient lactate production during myocardial ischemia [13], suggesting that PER2 protects the heart by regulating catabolism/energy. PER2 was also reported to be involved in regulating the cellular response to oxidative stress in mouse embryonic fibroblasts [14], pancreatic islets [15], and other organs. In the present study, we discovered that the circadian rhythm protein PER2 mediates susceptibility to I/R insult in a time-of-day-dependent fashion by regulating energy utilization and ROS clearance.

## 2. Methods

### 2.1. Animals

The use of specific pathogen-free C57BL/6J mice was approved by the Institutional Animal Care Committee at Zhujiang Hospital of Southern Medical University, China. Mice were housed in a temperature- and humidity-controlled specific pathogen-free facility with a 12 h light/dark cycle and *ad libitum* access to water and standard laboratory rodent chow.

### 2.2. Ischemia-Reperfusion (I/R) Surgery

I/R studies were performed on 10–12-week-old male mice. Mice were anesthetized with sodium pentobarbital (70 mg/kg intraperitoneally), placed on a temperature-controlled heating pad, and ventilated through endotracheal intubation. A standard 3-lead EKG and a rectal temperature probe were placed for monitoring. A thoracotomy was performed, and the heart was exposed by stripping the pericardium. The left anterior descending coronary artery was encircled by an 8-0 Prolene suture, and ischemia was induced by tightening the suture, which was confirmed visually by blanching of the distal cardiac tissue and ST-elevation on the electrocardiogram [16]. The suture was released after 30 min to allow reperfusion. The mice were maintained on ventilation until recovery from anesthesia. Infarct size was assessed 24 h postreperfusion using 1% Evans blue and 2% triphenyltetrazolium chloride (TTC) staining [17] and was expressed as a percentage of the at-risk area.

### 2.3. Echocardiographic Assessment

At the end of reperfusion, mice were reanesthetized with isoflurane, fixed on the experimental table, and studied with an echocardiography system (Vevo 2100, a high-resolution ultrasound echo-color Doppler system from VisualSonics, Canada). The following variables were measured and averaged during three consecutive cardiac cycles: left ventricular end-systolic diameter (LVESD), left ventricular end-diastolic diameter (LVEDD), left ventricular end-systolic volume (LVSV), and left ventricular end-diastolic volume (LVDV). The left ventricular ejection fraction (LVEF) and left ventricular fractional shortening (LVFS) values were converted by the Simpson method with the following formula: LVEF = (LVDV − LVSV)/LVDV × 100%; LVFS = (LVDD − LVSD)/LVDD × 100%. LVEF and LVFS were used as parameters indicating cardiac function [18]. The experiment was conducted three times, and the mean value was obtained.

### 2.4. Cell Culture and Serum Shock-Induced Circadian Rhythms

H9c2 cells were purchased from the National Collection of Authenticated Cell Culture (China). H9c2 cells were grown in an incubator in a DMEM-F12 medium (Corning) complemented with 10% fetal bovine serum (FBS; GIBCO) at 37°C in 5% CO_2_. Cells were subcultured every 4 days prior to the experiment in 6-well plates; they were confluent after 4 days. The cells were treated with 50% horse serum (50% DMEM-F12 and 50% horse serum, BI) for 2 h and were then washed twice with tepid DMEM-F12 without serum. Samples were taken every 4 h for 24 h following serum shock. Harvested cells were immediately frozen at −80°C until RNA isolation or protein extraction [19, 20].

### 2.5. H_2_O_2_ Treatment after Serum Shock in Cells Transfected with siRNA-Per2

For transfection experiments, 1 × 10^5^ H9c2 cells were plated in 6-well plates the day before transfection. Transfections were performed using 1 *μ*g siRNA oligo targeting *Per2* and 5 *μ*l Lipofectamine 3000 (Thermo Fisher, USA) in serum-free media per well, according to the manufacturer's protocol. After 12 h of transfection, the cells were treated with 50% horse serum for 2 h. At the end point or 12 h after serum shock, cells were treated with H_2_O_2_ for 2 h [21, 22].

### 2.6. TUNEL Staining

Cardiomyocyte apoptosis was assessed in heart sections by terminal deoxynucleotidyl transferase dUTP nick end labeling (TUNEL). The TUNEL mix (KeyGEN Biotech) contained 50 *μ*l enzyme solution and 450 *μ*l labeling solution. Heart sections were incubated with 50 *μ*l TUNEL mix at 37°C for 1 h. The sections were then washed twice with PBS and stained with DAPI. After washing with PBS for another three times, the sections were observed by fluorescence microscopy with Ex (*λ*) 450–500 nm, Em (*λ*) 515–565 nm for TUNEL and Ex (*λ*) 359 nm, Em (*λ*) 461 nm for DAPI (NIKON T12-E, Japan). The apoptosis ratio was calculated as apoptotic cell number (green)/total cell number (blue) × 100%.

### 2.7. Determination of ROS Production

Dihydroethidium (DHE, BestBio, China) staining was used to detect ROS levels in heart tissue. Fresh mouse heart tissue samples were embedded in an OCT compound (Thermo Fisher). Cryopreserved sections were then loaded with 500 *μ*M DHE following the manufacturer's instructions. Oxidized DHE was excited at 543 nm, and emission was collected with a LP 560 nm filter using the NIKON T12-E fluorescence microscope.

Intracellular ROS levels in H9c2 cells were determined by measuring the oxidative conversion of cell-permeable 2′,7′-dichlorofluorescein diacetate (DCFH-DA) to fluorescent dichlorofluorescein (DCF). The cells were washed with D-Hank's balanced salt solution (HBSS) and incubated with DCFH-DA at 37°C for 20 min. Then, fluorescence was detected by flow cytometry at Ex (*λ*) 488 nm, Em (*λ*) 525 nm (Beckman Coulter), which would collect and analyze ten thousand cells in each flow cytometric assay.

### 2.8. Determination of ATP Production

Adenosine 5′-triphosphate (ATP; Nanjing Jiancheng Bioengineering Institute, China) staining was used to detect ATP levels in heart tissue and H9c2 cells according to the manufacturer's instructions, using a microplate reader (Thermo Fisher).

### 2.9. Mitochondrial Membrane Potential (MMP) Measurement

The MMP of H9c2 cells was measured using the fluorescent probe, JC-1 (KeyGEN Biotech). The cells were rinsed with HBSS and incubated with JC-1 (10 *μ*M) at 37°C for 30 min. Afterwards, the cells were rinsed with HBSS once again. Fluorescent intensity of the JC-1 monomers and aggregates was detected by flow cytometry with Ex (*λ*) 490 nm, Em (*λ*) 530 nm for monomers and Ex (*λ*) 525 nm, Em (*λ*) 590 nm for aggregates (Beckman Coulter), and ten thousand cells were collected and analyzed each time.

### 2.10. Quantitative RT-PCR

Heart samples or H9c2 cells were disrupted/homogenized in a TRIzol reagent (Accurate Biotechnology, China) with a Tissue-lyzer (LUKYM, China), and total RNA was extracted according to the manufacturer's directions. RNA was reverse transcribed using the Accurate Biotechnology reverse transcription kit following the manufacturer's instructions. Quantitative PCR was performed using the SYBR Green Premix Pro Taq HS qPCR Kit (Accurate Biotechnology) using a two-step PCR amplification standard procedure on a Bio-Rad (USA) CFX connect system. Relative expression was calculated using the *ΔΔ*Ct method with normalization to *Gapdh*. Specific primer/probe sequences are shown below. Primer sequences are listed in Table 1 of supplementary data.

### 2.11. Immunoblots and Antibodies

Whole-cell lysates or isolated mitochondrial lysates were prepared by homogenizing the basal regions of the hearts in RIPA buffer (Invitrogen, USA) supplemented with protease 13 inhibitors (Invitrogen). Immunoblots were prepared using the antibodies listed below and were normalized to GAPDH (Sigma, USA). Antibodies used for western blots are listed in Table 2 of supplementary data.

### 2.12. Statistics

The results are presented as the means ± SEM. Two-tailed Student's *t*-tests were used to compare the difference between two groups. One-way or two-way ANOVA with Bonferroni correction was used for multiple comparisons. Statistical significance was defined as *P* < 0.05.

## 3. Results

### 3.1. Diurnal Differences in Myocardial Infarction Injury

**Figure 1:**
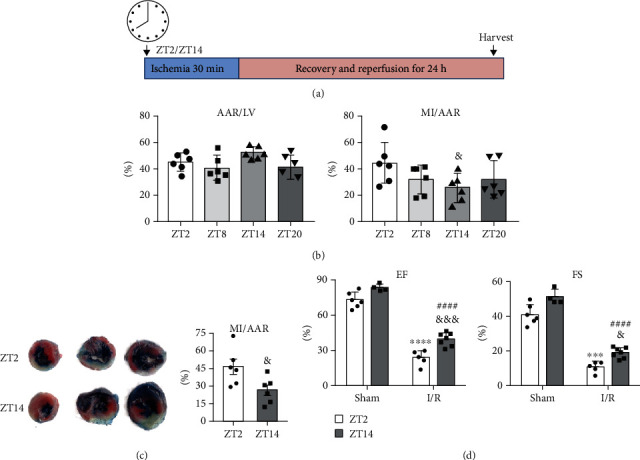
Circadian differences in susceptibility of the heart to I/R injury. (a) Experimental design. Mice were subjected to a protocol of 30 min of ischemia followed by 24 h of reperfusion starting at two time points, ZT2 or ZT14. ZT: zeitgeber time; ZT0: lights on. (b) Percent of area at risk over area of the left ventricle (middle). Percent of infarct over area at risk (right). AAR: area at risk; LV: left ventricle; MI: myocardial infarction (*n* = 6, ^&^*P* < 0.05 vs. ZT2). (c) Infarct size. Representative Evans blue and TTC staining (left). Percent of infarct over area at risk (right). White: infarct; red: viable; white+red: area at risk; blue: retrograde Evans blue staining, area not at risk (*n* = 6, ^&^*P* < 0.05 vs. IR-ZT2, two-tailed Student's *t*-test). (d) Cardiac systolic function of mice with I/R at ZT2 and ZT14. EF of the left ventricle (left), FS of the left ventricle (right). EF: ejection fraction; FS: fraction shortening; sham: sham surgery; I/R: ischemia-reperfusion. (*n* = 4–7, ^∗∗∗∗^*P* < 0.0001 and ^∗∗∗^*P* < 0.001 vs. sham-ZT2; ^####^*P* < 0.0001 vs. sham-ZT14; ^&^*P* < 0.05 and ^&&&^*P* < 0.001 vs. IR-ZT2, two-tailed Student's *t*-tests). Alpha was set as 0.05. Data are presented as the mean ± SEM. The Holm-Sidak method was used to correct for multiple *t*-tests.

The infarct area of cardiac tissue in each group was detected by TTC and Evans blue staining and quantified using ImageJ. The results showed that the area at risk in the left ventricle (AAR/LV) after I/R was not significantly different among the four groups (Figure 1(b), left). However, the infarct area in the area at risk (IR/AAR) after I/R at ZT2 was larger than at ZT14, while no difference was observed at ZT8 or ZT20 (Figure 1(b), right). As shown in Figure 1(c), ischemia followed by reperfusion induced significant myocardial injury as denoted by the white infarct size in mice. In contrast, ZT14 significantly decreased the ratio of infarct area/risk area (MI/AAR) compared with the ZT2 group (Figure 1(c), right).

M-mode ultrasound was used to evaluate changes in cardiac function of 10~12-week-old mice in each group, 24 h after myocardial I/R. Typical M-mode ultrasounds showed that compared with the sham group without I/R at zeitgeber time (ZT) 2 or ZT14, the ejection fraction (EF) of the left ventricle was decreased in mice 24 h after I/R. In addition, the EF in mice after I/R at ZT2 was lower than that in mice after I/R at ZT14 Figure 1(d), left). At the same time, fraction shortening (FS) of the left ventricle in mice after I/R at ZT2 was lower than that in mice with I/R at ZT14 (Figure 1(d), right), which showed that mice receiving I/R at ZT2 had worse cardiac systolic function than at ZT14.

### 3.2. Diurnal Differences in Oxidative Stress and Energy Metabolism

**Figure 2:**
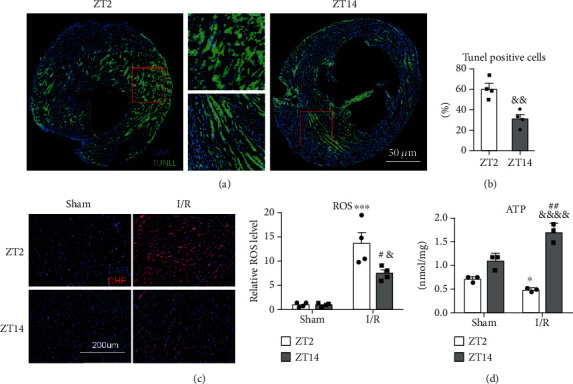
Circadian differences in oxidative stress and energy metabolism. (a) Apoptotic cells were determined by TUNEL staining. Representative apoptotic cells of mouse hearts after I/R injury at ZT2 (left) and ZT14 (right). Magnified area of interest from mouse heart after I/R injury at ZT2 (up middle) and ZT14 (bottom middle). (b) Quantitative analysis of TUNEL cells (*n* = 4, ^&&^*P* < 0.01 vs. IR-ZT2, two-tailed Student's *t*-test). (c) ROS in the hearts of mice. Representative DHE staining of the peri-infarct area of mouse hearts after 24 h IR at ZT2 and ZT14 (left), quantitative analysis of ROS (middle) (*n* = 4, ^∗∗∗^*P* < 0.001 vs. sham-ZT2; ^#^*P* < 0.05 vs. sham-ZT14; ^&^*P* < 0.05 vs. IR-ZT2, two-tailed Student's *t*-tests). (d) ATP change in mouse hearts with I/R injury (*n* = 3, ^∗^*P* < 0.05 vs. sham-ZT2; ^##^*P* < 0.01 vs. sham-ZT14; ^&&&&^*P* < 0.0001 vs. IR-ZT2, two-tailed Student's *t*-tests). Alpha was set as 0.05. Data are presented as the means ± SEM. The Holm-Sidak method was used to correct for multiple *t*-tests.

Histological analysis and immunofluorescence staining were performed to further evaluate myocardial cell activity. Typical results of TUNEL staining showed that cell apoptosis of myocardial tissue after I/R at ZT2 was more extensive than that observed in the ZT14 group (Figure 2(a)). These results suggested that I/R at ZT2 could significantly increase myocardial cell apoptosis (Figure 2(b)).

DHE staining results showed that ROS was increased in myocardial cells after I/R, where the ROS level in the ZT2 group was higher than that in the ZT14 group (Figure 2(c)).

ATP determination assays performed in each group showed that without I/R, sham-ZT14 mice produced more ATP than sham-ZT2 mice, although the difference was not statistically significant. In contrast, 24 h after I/R at ZT2, ATP content was lower than in sham-ZT2 mice. The ATP content 24 h after I/R at ZT14 was higher than that in the sham-ZT14 group, which indicated that mice produced more ATP at ZT14 while producing less ATP at ZT2 (Figure 2(d)).

### 3.3. Diurnal Differences Affected the Expression of PER2 and CPT1A

**Figure 3:**
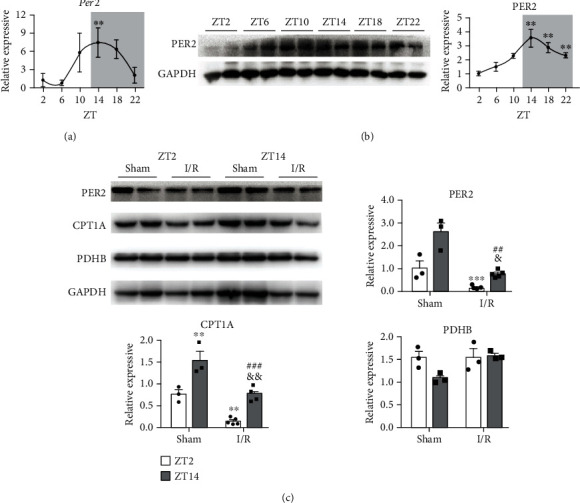
PER2 in circadian I/R susceptibility of the heart. *Per2* mRNA (a) and (b) protein expression in mouse heart during a 24 h day. Quantification was normalized to GAPDH. ZT2 was set to 1 (*n* = 3 per time point, ^∗∗^*P* < 0.01 and ^∗∗∗^*P* < 0.001 vs. ZT2; one-way ANOVA with Bonferroni correction). (c) PER2 and potential target levels were determined by immunoblots with or without I/R injury at ZT2 and ZT14. Quantification of PER2, CPT1, and PDHB was normalized to GAPDH (*n* = 3–5, ^∗∗^*P* < 0.01 and ^∗∗∗^*P* < 0.001 vs. sham-ZT2; ^##^*P* < 0.01 and ^###^*P* < 0.001 vs. sham-ZT14; ^&^*P* < 0.05 and ^&&^*P* < 0.01 vs. I/R-ZT2, two-tailed Student's *t*-test). Alpha was set as 0.05. Data are presented as the means ± SEM. The Holm-Sidak method was used to correct for multiple *t*-tests.

mRNA and protein were collected from the mice every 4 h for 24 h. The transcription level of *Per2* showed a distinct rhythmic pattern for nearly 24 h (Figure 3(a)). The transcription level of *Per2* increased from ZT2, reaching a peak value near ZT14, then decreased again to a low value near ZT2 (Figure 3(a)). The change in PER2 protein levels was consistent with the mRNA results. Mice displayed a rhythm in PER2 protein expression with peak values at ZT14 and lowest values at ZT2 as shown by western blotting (Figure 3(b)). The protein expression of PER2, carnitine palmitoyltransferase 1A (CPT1A), and pyruvate dehydrogenase PDH-E1B (PDHB) after I/R was also detected by western blotting (Figure 3(c)). The results showed that the protein expression of PER2 and CPT1A was similar under the same treatment conditions and decreased after I/R at ZT2, while increasing after I/R at ZT14. PDHB showed no difference after I/R.

### 3.4. Downregulation of PER2 Increased Injury

**Figure 4:**
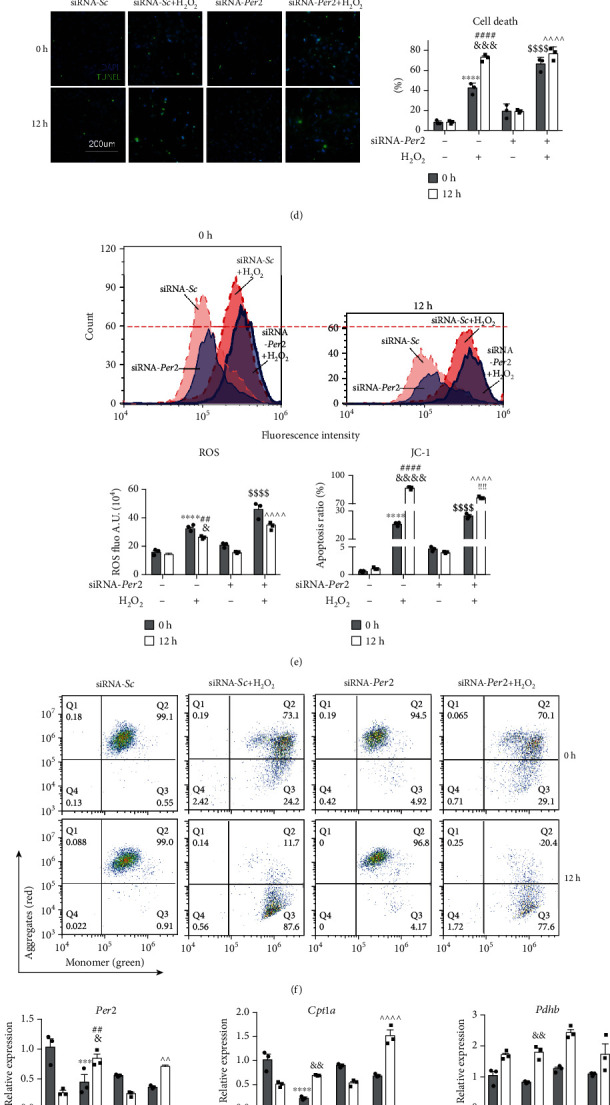
PER2 deficiency in the tolerance of H9c2 cells to H_2_O_2_ treatment after serum shock. (a) *Per2* mRNA expression during 24 h after serum shock (*n* = 3 per time point, ^∗^*P* < 0.05 vs. 0 h after serum shock, one-way ANOVA with Bonferroni correction). (b) *Per2* mRNA expression levels in H9c2 cells after knockdown of *Per2* using siRNA (*n* = 3, ^∗∗∗^*P* < 0.001 vs. siRNA-Sc-0 hr, ^#^*P* < 0.05 vs. siRNA-Sc-12 hr). (c–g) H9c2 cells were transfected with siRNA-*Sc* or siRNA-*Per2* 24 h before serum shock. Transfected cells were treated with H_2_O_2_ for 2 h at the ending point or 12 h after serum shock. (c) ATP change in transfected H9c2 cells treated with H_2_O_2_ at 0 h and 12 h after serum shock. (d) Cell death determined by TUNEL staining. Representative image (left) and quantification (right). (e) ROS in cells. Flow cytometry images (upper), quantitative analysis of ROS (lower panel, left figure). (f) Flow cytometry of JC-1 in cells. Representative images (f), quantitative analysis of JC-1 ((e), lower panel, right figure). (g) *Per2* and potential target levels in H9c2 cells were determined by RT-PCR at 0 h and 12 h. Quantification of *Per2*, *Cpt1a*, and *Pdhb* was normalized to GAPDH (*n* = 3, ^∗∗^*P* < 0.01, ^∗∗∗^*P* < 0.001, and ^∗∗∗∗^*P* < 0.0001 vs. cells transfected with siRNA-*Sc* at 0 h after serum shock; ^#^*P* < 0.05, ^##^*P* < 0.01, and ^####^*P* < 0.0001 vs. cells transfected with siRNA-*Sc* at 12 h after serum shock; ^&^*P* < 0.05 and ^&&&&^*P* < 0.0001 vs. cells transfected with siRNA-*Sc* treated with H_2_O_2_ at 0 h after serum shock; ^$$$$^*P* < 0.0001 vs. cells transfected with siRNA-*Sc* with H_2_O_2_ treatment at 0 h after serum shock; ^^^^^^*P* < 0.0001 vs. cells transfected with siRNA-*Sc* with H_2_O_2_ treatment at 12 h after serum shock; ^!!!!^*P* < 0.0001 vs. cells transfected with siRNA-*Per2* with H_2_O_2_ treatment at 0 h after serum shock). Alpha was set as 0.05. Data are presented as the means ± SEM. The Holm-Sidak method was used to correct for multiple *t*-tests.

H9c2 cells that were transfected with siRNA-*Per2* were collected after H_2_O_2_ treatment and serum shock at 0 h and 12 h. mRNA was collected every 4 h for 24 h. The transcription level of *Per2* showed a distinct rhythmic pattern for nearly 24 h (Figure 4(a)). From 0 h after serum shock, the transcription level of *Per2* decreased until reaching a low value near 12 h (Figure 4(a)). After transfection with siRNA-Per2, there was a significant reduction in PER2, shown in Figure 4(b).

ATP determination assays were also performed for each group. After H_2_O_2_ treatment, ATP was decreased at 12 h but was increased at 0 h (Figure 4(c)). ATP levels were significantly decreased after siRNA-*Per2* transfection (Figure 4(c)), suggesting that downregulation of PER2 decreased ATP.

Histological analysis and immunofluorescence staining were performed to further evaluate H9c2 cell activity. The results of TUNEL staining showed that cellular apoptosis of H9c2 cells after H_2_O_2_ treatment at 0 h was less than apoptosis at 12 h after serum shock (Figure 4(d)). After siRNA-*Per2* transfection, cell death increased compared with the control group, but there was no significant difference at 0 h or 12 h after serum shock whether the cells were treated with H_2_O_2_ or not. These results indicated that in H9c2 cells, 12 h of serum shock and siRNA-*Per2* transfection could both increase myocardial cell apoptosis (Figure 4(d)).

ROS detected by flow cytometry showed that in cells treated with H_2_O_2_, ROS increased significantly (Figure 4(e)). At the same time, ROS increased after siRNA-*Per2* transfection, whether or not the cells were treated with H_2_O_2_ (Figure 4(e)).

Flow cytometric analysis was performed to identify quantitatively the MMP in H9c2 cells, which is one of the important hallmarks of mitochondrial damage. The contour plots in Figure 4(f) showed that the fluorescence for high MMP in Q2 was shifted to low MMP in Q3. H_2_O_2_ treatment showed a strong ability to dissipate the MMP, and cells after 12 h of serum shock showed more dissipation of MMP than cells at 0 h (Figure 4(f)). Furthermore, transfection of siRNA-*Per2* resulted in more dissipation of MMP. These results suggested that the mitochondrial membrane was impaired by H_2_O_2_, siRNA-*Per2* transfection, and 12 h of serum shock (Figure 4(e), lower panel, right figure).

mRNA expression of *Per2*, *Cpt1a*, and *Pdhb* in H9c2 cells was quantified by RT-PCR analysis (Figure 4(g)). The results showed that the expression of *Per2* and *Cpt1a* was similar under the same treatment conditions. Expression of these genes was higher before serum shock compared with 12 h later and decreased after H_2_O_2_ treatment or siRNA-*Per2* transfection.

## 4. Discussion

Taken as a whole, the current findings suggested that PER2 was the basis of circadian changes in cardiac tolerance to I/R and that knockdown of PER2 expression could increase I/R damage. Furthermore, PER2 likely acted downstream as a mediator underlying the transcriptional clock mechanism in cardiac susceptibility to I/R damage by regulating CPT1A as a key enzyme in fatty acid metabolism and oxidative stress levels. This result is important because it focuses on energy metabolism and reactive oxygen species as potential therapeutic targets, which represent physiological oscillations and metabolic changes in the cardiovascular system itself as the basis for cardiac protection.

Many studies have implicated circadian rhythm in the brain [23], liver [24], lung [25], kidney [26], and heart [27]. Epidemiologic studies have reported circadian rhythmicity in the incidence and the size of myocardial infarctions which were higher during the day and lower at night [28]. In addition to external factors such as neurohormonal regulation, platelet aggregation, and vascular activities, the vulnerability of the myocardium shows a diurnal variation, but the molecular mechanism is not clear.

In this study, we found that mice showed a bigger infarct at ZT2 (Figure 1) and were susceptible to cardiac I/R damage at the end of the light phase. This result was similar to a previous basic research report of Li et al. [7], who suggested that a deficiency of the oscillating transcription factor KLF15 may specifically cause NAD+ deficiency during the sleep-to-active transition and may increase the susceptibility of the heart muscle to I/R injury. Interestingly, other studies have shown opposite results, with ZT14/ZT12 having larger infarcts than ZT2/ZT0 [29]. As Rotter et al. reported, cardiac damage from ischemia/reperfusion was greatest at the transition from sleep to activity, and Rcan1 control of calcineurin activation was necessary for the daily oscillations of the heart against injury [30]. However, Rcan1 KO was equally more susceptible to damage from I/R at the end of the light phase. We hypothesized that these differences in results might be related to the timing of ischemia and reperfusion, reflecting the fundamental difference in the cell injury mechanism between ischemia and reperfusion.

Combined with previous studies, diurnal variations in the onset of myocardial infarction are well established. The influence of the circadian clock cannot be ignored in heart I/R studies. Various proteins compose the circadian clock, including PER2, CRY, CLOCK, and BMAL [31]. PER2 can “buffer” or adjust the antagonism between CLOCK/BMAL1 and CRY, so that CLOCK reaches a self-limiting, rhythmic cycle [32]. Through RT-PCR and western blots, we saw that in C57BL/6 mice, PER2 expression showed a distinct rhythmic pattern for nearly 24 h (Figure 3), and the valley of PER2 expression coincided with the time when the damage resistance of the heart was reduced after the transition from dark to light. Another study has shown that mice with *Per2-/-* had enhanced tissue damage from myocardial ischemia and lacked the capacity to enhance oxygen-efficient glycolysis [33].

Our results suggested that the absence of PER2 was associated with mitochondrial injury, which then led to less ATP and more ROS production, thus increasing cell injury. After cardiac ischemia-reperfusion, the oxidative and antioxidant capacities of myocardial cells are unbalanced, resulting in a state of “oxidative stress,” which plays an important role in the injury caused by myocardial infarction. Reactive oxygen species and oxidative stress have been reported to play important roles in the progression after myocardial infarction [34]. Wu et al. demonstrated that acacetin stimulated AMPK, which mediates activation of the Nrf2 signaling pathway in cardiomyocyte protection against hypoxia/reoxygenation injury by increasing the antioxidants heme oxygenase 1 (HO-1), SOD1, and SOD2, reducing ROS production and thereby effectively inhibiting hypoxia/reoxygenation injury [35]. As reported by Ortiz et al., the use of thyroid hormone (TH) as a therapeutic alternative revealed cardioprotective effects after AMI, including decreased oxidative stress, while carvedilol and TH coadministration improved redox balance and cardiac function after AMI [36]. Finding a promising drug which protects cardiomyocytes against hypoxia/reoxygenation may help manage ischemic cardiac disorders.

To pinpoint mitochondrial processes that may be under circadian gene PER2 control, we examined two central mitochondrial metabolic pathways of carbohydrate metabolism and fatty acid uptake. The rate-limiting step in mitochondrial carbohydrate metabolism is carried out by the PDC, a multiprotein complex that catalyzes the oxidative decarboxylation of pyruvate [37]. We found that several components of the PDC, namely, the catalytic pyruvate dehydrogenase PDHB, PDH-E2 (DLAT), and the regulatory subunit PDHX, were involved. The rate-limiting step for the entry of long-chain fatty acids into the mitochondrial matrix is the synthesis of acylcarnitine from acyl CoA and carnitine, which is mediated by CPT1A [38]. It has been reported that the mRNA levels of *Pdhb* and *Cpt1a* also cycle for 24 h but reached their peak levels, respectively, at ZT16 and ZT17 [39]. Our results showed that the expression of *Per2* and *Cpt1a* was basically similar, and peak expression of *Per2* and *Cpt1a* coincided with the time when damage resistance of the heart was increased after the transition from light to dark, while that of *Pdhb* was different (Figure 4(g)). This indicated that PER2 damage to cardiomyocytes was mediated by CPT1A, which produced less ATP and provided insufficient energy to cardiomyocytes, thus leading to the aggravation of myocardial injury.

At present, the mechanisms linking circadian rhythm stabilization and myocardial infarction are under investigation and could involve inflammation [40], glycolysis metabolism [41], cyclin-dependent kinase 5 [42], or adenosine [33]. Some of these are known to be associated with PER2 and contribute to the survival of the myocardium after I/R. Sun et al. hypothesized that *Per2* deficiency affected endothelial progenitor cell function through CXCR4/PI3k/Akt/FoxO-related mechanisms involving angiogenesis in the ischemic myocardium of mice, thus increasing damage [43]. Kobayashi et al. established the dependence of HIF1A on PER2 as a transcription factor during hypoxia and proposed that PER2 acted as an effector molecule for the recruitment of HIF1A to promoter regions of its downstream genes [44]. On this basis, Oyama et al. also found that specific HIF1A pathways controlled glycolysis, mitochondrial respiration (COX4.2), or endothelial barrier function (ANGPTL-4/claudin-1) after PER2 overexpression [41].

Numerous studies have demonstrated links between myocardial infarction and disruption of circadian homeostasis both in clinical observations and in basic research findings. Therefore, it is important to maintain the homeostasis of circadian rhythms in humans. Interestingly, in addition to the above studies, chronic shift work [45], anesthetics [46], melatonin [47], and intestinal microbiota [48] can also affect circadian rhythms.

In summary, we have shown that the injury caused by myocardial infarctions has circadian rhythmicity. Our study suggests that PER2 deficiency contributed to the susceptibility of the myocardium to I/R injury, with peak susceptibility in the early morning, through reactive oxygen species and CPT1A as a key enzyme in energy metabolism, which may be particularly harmful to patients during a critical time window.

## Data Availability

Data are available on request to the authors.

## Supplementary Materials

Supplementary Materials TABLE 1: primer sequences used in reverse transcription-polymerase chain reactions. TABLE 2: antibodies used for western blots.
