# Supplementary material for: PER2 Regulates Reactive Oxygen Species Production in the Circadian Susceptibility to Ischemia/Reperfusion Injury in the Heart
Source: Oxid Med Cell Longev. 2021 Oct 8;2021:6256399. doi: 10.1155/2021/6256399 (PMC8519710; doi:10.1155/2021/6256399)
Supplement: Supplementary Materials — TABLE 1: primer sequences used in reverse transcription-polymerase chain reactions. TABLE 2: antibodies used for western blots. [file 6256399.f1.docx]

Supplementary data

Table 1: Primer sequences used in reverse transcription–polymerase chain reactions.

| **Primer name** | **Target species** | **Sequence**  **5eque** |
| --- | --- | --- |
| *Per2* | mouse | F-TGACGCACACAAAGAACTGA R-CCCTTGGCCTTCTTGTCTG |
| *Gapdh* | mouse | F-GGTTGTCTCCTGCGACTTCA R-TGGTCCAGGGTTTCTTACTCC |
| *Cpt1a* | mouse | F-CACCAACGGGCTCATCTTCT R-CCTTCTATCGAATTTGCTCTGGTT |
| *Pdhb* | mouse | F-TTAAATCGGCCATTCGTGAT R-CAGGAAATCTTTTGACTGAGCTT |
| *Per2* | Rat | F-CATCTGCCACCTCAGACTCA R-CTGGTGTGACTTGTATCACTGCT |
| *Gapdh* | Rat | F-GACATGCCGCCTGGAGAAAC R-AGCCCAGGATGCCCTTTAGT |
| *Cpt1a* | Rat | F-CATTACAAGGACATGGGCAAGTT R-CGGAAGAGCCGGGTCAT |
| *Pdhβ* | Rat | F-CCCGGTTTGAAAGTGGTCAG R-ACTACAGTGATGTGGGTCCC |

Table 2: Antibodies used for western blots.

| Antibody | Source |
| --- | --- |
| Rabbit polyclonal anti-PER2 antibody | Thermo Fisher Scientific |
| Mouse monoclonal anti-GAPDH antibody | Beijing Ray Antibody Biotech |
| Rabbit monoclonal anti-CPT1A antibody | Abcam |
| Rabbit monoclonal anti- PDHB antibody | Abcam |
| Goat anti-mouse IgG secondary antibody | Abcam |
| Goat anti-rabbit IgG secondary antibody | Abcam |
